# Characteristics and Outcomes in Primary Aldosteronism Patients Harboring Glucocorticoid-Remediable Aldosteronism

**DOI:** 10.3390/biomedicines9121816

**Published:** 2021-12-02

**Authors:** Chung-Yi Cheng, Hung-Wei Liao, Kang-Yung Peng, Tso-Hsiao Chen, Yen-Hung Lin, Jeff S. Chueh, Vin-Cent Wu, on behalf of the TAIPAI Study Group

**Affiliations:** 1 Division of Nephrology, Department of Internal Medicine, School of Medicine, College of Medicine, Taipei Medical University, No. 250 Wu-Hsing Street, Taipei 110, Taiwan; 94426@w.tmu.edu.tw (C.-Y.C.); 88128@w.tmu.edu.tw (T.-H.C.); 2 Division of Nephrology, Department of Internal Medicine, Wan Fang Hospital, Taipei Medical University, No. 111 Section 3, Xinlong Road, Taipei 116, Taiwan; 3 Taipei Medical University-Research Center of Urology and Kidney (RCUK), School of Medicine, College of Medicine, Taipei Medical University, No. 250 Wu-Hsing Street, Taipei 110, Taiwan; 4 Chinru Clinic, Taipei 116, Taiwan; lhw898@gmail.com; 5 Department of Internal Medicine, National Taiwan University Hospital, Room 1555, Clinical Research Building, 7 Chung-Suan South Road, Taipei 100, Taiwan; kangyung@ntu.edu.tw (K.-Y.P.); yenhunglin@ntuh.gov.tw (Y.-H.L.); 6 Department of Urology, College of Medicine, National Taiwan University, National Taiwan University Hospital, Taipei 110, Taiwan; scchueh@ntu.edu.tw

**Keywords:** glucocorticoid-remediable aldosteronism, hypokalemia, plasma renin activity, adrenalectomy

## Abstract

The clinical characteristics and surgical prognosis of glucocorticoid-remediable aldosteronism (GRA, also known as familial hyperaldosteronism type 1, FH-I) have not been widely studied. Using data from the Taiwan Primary Aldosteronism Investigation (TAIPAI) registry retrospectively, we describe the associated clinical factors for GRA and clinical predictors of surgical outcomes among identified GRA patients. We found 79 GRA-positive (51.2 ± 13.8 years; women 39 (49.4%)) and 114 GRA-negative primary aldosteronism (PA) patients matched with age, gender, and body mass index. Lower plasma aldosterone concentrations (PACs) and aldosterone-renin ratios were found among GRA-positive individuals. Multivariable logistic regression demonstrated that a PAC ≤ 40 ng/dL could predict concealed GRA individuals (OR 0.523, *p* = 0.037). Low serum potassium (OR 0.285, *p* = 0.008), but not the presence of GRA, was associated with hypertension-remission. Of note, PRA (OR 11.645, *p* = 0.045) and hypokalemia (OR 0.133, *p* = 0.048) were associated with hypertension-remission in GRA patients. Unilateral primary aldosteronism patients harboring concomitant GRA were not associated with inferior hypertension-remission after an adrenalectomy. Low serum potassium and high PRA were positively associated with hypertension-remission in GRA patients.

## 1. Introduction

Familial hyperaldosteronism type 1 (FH-I) is a hereditary form of primary aldosteronism (PA) due to chimeric gene duplication that leads to ectopic aldosterone synthase activity. FH-I is also known as glucocorticoid-remediable aldosteronism (GRA), where the adrenocorticotropic hormone (ACTH) controls aldosterone secretion. GRA appears to be the most common monogenic cause of hypertension in humans [1,2,3]. The classical characteristic of GRA is mineralocorticoid excess, including hypertension, hypokalemia, and suppressed plasma renin activity (PRA). In 1966, Sutherland and colleagues described the reversible nature of GRA by administering exogenous glucocorticoids such as dexamethasone [4]. Recent advances in molecular biology have led to a better understanding of the genetic characteristics of GRA. 

The most common presentation of GRA is the discovery of severe asymptomatic hypertension, especially in infancy and early adulthood [5,6]. There is an increased frequency of early death from stroke in GRA-affected families, and an increased risk of preeclampsia during pregnancy [7,8,9]. However, some GRA patients may only be mildly hypertensive or even normotensive [10]. The variability in blood pressure readings in GRA patients may be related to other hereditary factors that regulate blood pressure or environmental factors, such as variation in dietary sodium intake [11,12]. Although GRA is a state of mineralocorticoid excess, clinically significant hypokalemia is uncommon [7]. Biochemically, in GRA aldosterone secretion is regulated by ACTH, which is not sensitive to sodium homeostasis, resulting in hyperaldosteronism and volume expansion. As a result, renin activity is suppressed by hyperaldosteronism. 

Historically, it is believed that GRA accounts for about 1% of all PA patients. The current clinical practice guidelines recommend the use of genetic screening for GRA in patients with confirmed PA who had hypertension onset at the age of 20 or earlier, who have a family history of PA, or who had a stroke at 40 years of age or less [13]. The value of clinical variables that predict the diagnosis of GRA remains to be elucidated. The surgical benefit and contributory factors to surgical outcomes among GRA patients have not been well discussed either. 

The present study retrospectively assessed the correlation between biochemical and clinical parameters in uPA patients harboring GRA. Moreover, we also evaluated clinical and biochemical success after treatment using the up-to-date outcome criteria. 

## 2. Materials and Methods

### 2.1. Ethical Approval

This study complied with the Declaration of Helsinki and was approved by the National Taiwan University Hospital Research Ethics Committee (No. 200611031R, 16 June 2017). All participants received comprehensive written information and signed a consent form before their inclusion in this study. Moreover, the genetic testing was also approved by the Taiwan University Hospital Research Ethics Committee. 

### 2.2. Study Design and Participants

The inception cohort was based on the Taiwan Primary Aldosteronism Investigation (TAIPAI) database and tissue bank. The TAIPAI registry was constructed for quality assurance in two medical centers, three affiliated hospitals, and two regional hospitals in different cities of Taiwan [13]. Antihypertensive medications were discontinued for at least 21 days before confirmatory tests. When necessary, diltiazem and/or doxazosin were administered to control markedly high blood pressure [14]. Patients with an initial aldosterone-renin ratio (ARR) > 35 were confirmed as PA by saline infusion or captopril tests. Subtypes of PA were identified via imaging. Criteria for unilateral primary aldosteronism (uPA) identification were [14]: (1) confirmed PA diagnosis; (2) image evidence for a unilateral adrenal adenoma or hyperplasia; (3) lateralization of aldosterone secretion with adrenal vein sampling (AVS) [15] or during dexamethasone suppression NP-59 SPECT/CT to the above-mentioned image-finding side; uPA was further confirmed after an adrenalectomy with (4) a pathologically proven CYP11B2-positive stained adenoma [16,17] or immunohistochemical evidence for (multiple) aldosterone-producing micronodule(s)/nodules ((M) APM/APN) after an adrenalectomy.

For this study, uPA patients, matched by age, sex, and body mass index (BMI), who had negative genetic screening results for GRA were selected for the control group; thus, GRA-negative patients were defined as uPA patients with negative GRA gene expression by long-range PCR.

### 2.3. Diagnosis of GRA Using Long-Range PCR

A modified version of long-range polymerase chain reaction (PCR) using the method described by MacConnachie et al. was performed to identify the chimeric gene [14,15]. In brief, the primer sets of forward and reverse sequences listed below were used to amplify the normal CYP11B2 gene and the chimeric CYP11B1/CYP11B2 gene with PfuUltra II Fusion HS DNA polymerase (Agilent). The touchdown PCR process used the following protocol: 95 °C for 2 min; 38 cycles of denaturation at 95 °C for 1 min, annealing at 70–61 °C for 1 min, and extension at 72 °C for 5 min; 72 °C for 3 min. The annealing temperature began at 70 °C and lowered 1 °C every two cycles until it reached 61 °C. This annealing temperature was maintained until the end of the cycling process. The long-range PCR amplicon was analyzed in a 0.8% agarose gel, cleaned up with the 0.45X Agencourt AMPure XP beads (Beckman Coulter), and quantified using a QubitTM fluorometer (Life Technologies). Primer sets of the CYP11B2 gene, as per our report, were [16]: Forward sequence 5′CAGGTCCAGAGCCAGTTCTCCCAT and reverse sequence 5′ACCCTCCTTCTCCTTGTACACCCA. Primer sets of the CYP11B1/CYP11B2 gene, as per our report, were [16]: Forward sequence 5′CAGTTCTCCCATGACGTGATCCCT and reverse sequence 5′ACCCTCCTTCTCCTTGTACACCCA. The expected size of the chimeric gene and the uncropped Southern blot image of the long-range PCR are shown in the Supplementary Materials, Figure S1.

### 2.4. Outcomes of Interest

We defined the clinical and biochemical outcomes according to the consensus of the Primary Aldosteronism Surgical Outcome (PASO) with at least two follow-up visits [17,18].

### 2.5. Statistical Analysis

Data were expressed as mean ± standard deviation unless otherwise specified. Stepwise binary logistic regression analysis was used to investigate the relationships between biochemical and clinical risk factors for GRA. The backward stepwise regression method was used to select variables in the multivariate analysis. The logarithmic scales of plasma aldosterone concentration (PAC), plasma renin activity (PRA), and aldosterone/renin ratio (ARR) were selected for multivariate analysis in order to avoid errors being generated due to the collinearity PRA and ARR. Differences between the means of multiple subgroups were assessed using the Kruskal–Wallis test. A generalized additive model (G.A.M.) (with spline) incorporated subject-specific random effects, expressed as the logarithm of the odds (logit), and the optimal cut-off value was defined as a log-odds value of zero [19,20,21]. An unpaired t-test or the Mann–Whitney U test was used for continuous variables. All analyses were performed with R software, version 3.2.2 (Free Software Foundation, Inc., Boston, MA, USA) [22], and SPSS version 25 (SPSS Inc., Chicago, IL, USA) was also used for analysis. A *p*-value of < 0.05 was considered statistically significant.

## 3. Results

### 3.1. Clinical Characteristics of uPA Patients

A total of 193 patients diagnosed with uPA were included in this study. There were 79 GRA-positive patients, and they were matched with 114 GRA-negative uPA patients according to age, gender, and BMI (Table 1). Moreover, GRA patients had significantly lower PAC and ARR than the GRA-negative patients.

### 3.2. Factors Associated with GRA

The association of clinical and biochemical parameters for GRA was examined by univariable and multivariable logistic regression (Table 2). Both age (OR 1.02, 95% CI 1.00–1.04) and eGFR (OR 1.01, 95% CI 0.98–1.02) were positively associated with GRA. In contrast, plasma aldosterone was negatively associated with GRA (OR 0.16, 95% CI 0.05–0.51).

**Figure 1:**
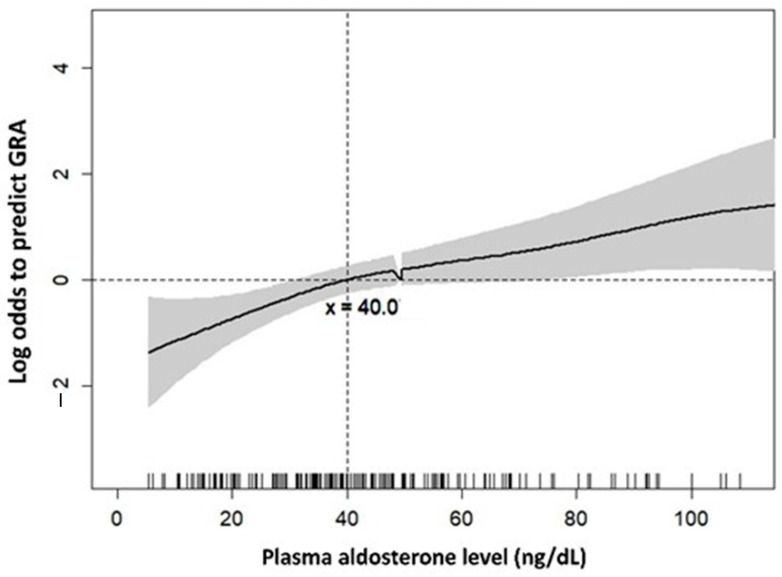
Generalized additive model (GAM) plot for plasma aldosterone and log odds to predict GRA. Figure 1 depicts the GAM plot for the probability of plasma aldosterone against GRA-positive patients, incorporating the subject-specific (longitudinal) random effects expressed as the logarithm of the odds. The probability of outcome events was constructed with age-adjusted variables over the range of data, and plasma aldosterone = 40. The dashed lines represent approximated pointwise 95% CI. Dotted curves indicate 95% CIs for the smoothed hazard.

A GAM plot was used to find the adequate cut-point value for the plasma aldosterone level to predict the probability of GRA. At the cut-point of 40 ng/dL, plasma aldosterone negatively predicted GRA after adjusting the age of GRA individuals (Figure 1).

### 3.3. Lower Potassium Associated with Better Surgical Outcomes in All PA Patients

In this study, 97 unilateral PA (uPA) patients received an adrenalectomy, including 36 GRA and 61 GRA-negative patients. According to the criteria established by the Primary Aldosteronism Surgical Outcome (PASO) consensus [18], 12 of the 36 (33.3%) GRA and 22 of the 61 (36.1%) GRA-negative patients were considered to be surgically successful after the adrenalectomy (Table 3). The lower serum potassium levels had better hypertension-remission among uPA patients (OR 0.285, 95% CI 0.113–0.717, *p* = 0.008) (Table 3). The presence of GRA was not associated with surgical outcomes (*p* = 0.638).

### 3.4. Lower Potassium and High PRA Associated with Better Surgical Outcomes in GRA Patients

After adjusting the variables, serum potassium was found to be negatively associated with surgical success (OR 0.133, 95%CI 0.018–0.978, *p* = 0.048) in GRA patients. In contrast, PRA was found to be positively associated with hypertension-remission among GRA patients (OR 11.6, 95%CI 1.1–128.9, *p* = 0.045) (Table 4).

## 4. Discussion

This study revealed that uPA and GRA could occur concurrently in patients, and those uPA patients with concomitant GRA had significantly lower plasma aldosterone and lower ARR than GRA-negative patients. Moreover, the multivariable analysis showed that the older age and better eGFR in the uPA patients appeared to be positive associated with GRA. Lower serum potassium and higher PRA were positively associated with hypertension-remission in uPA patients harboring GRA.

Several studies have suggested that PA is the most common cause of secondary hypertension, with the estimated prevalence range being from 1.4% to 32% for all forms of hypertension [23,24]. Considering the phenotypic variabilities found among genetically affected GRA, the actual prevalence of GRA among the hypertensive population still needs to be established. Although genetic testing is highly sensitive and specific, screening for the presence of GRA in every hypertensive patient may not be clinically feasible. Genetic testing for GRA has been suggested for patients who had an onset of confirmed PA before 20 years of age and those who have a family history of PA or stroke at a young age [13]. The present study and logistic regression showed that age was negatively associated with GRA. The TAIPAI registry further documented that GRA patients were associated with kidney function impairment. Quantitative histopathological analysis revealed that PA patients had significantly larger glomerular sizes, interstitial fibrosis, and more pronounced arteriolar hyalinization [25]. These pathological findings suggested that the process of glomerular hyperfiltration led to progressive renal impairment in PA patients [25]. The development of progressive kidney impairment in PA patients was probably initiated by glomerular hyperfiltration [19]. We endorsed that young PA patients with chronic kidney disease should have their genetic testing carried out for GRA based on such evidence.

### 4.1. PA Patients Harboring GRA and Clinical Outcome

The rate of complete clinical success after a unilateral adrenalectomy in uPA patients varied from 9.6% to 45.7% [20,21,22,26]. In this study, 35% of uPA patients achieved complete clinical success (33.3% GRA-positive vs. 36% GRA-negative PA patients). We found that the surgical outcome was not associated with the presence or absence of GRA status by logistic regression. The dietary habit might contribute to PA patients with different surgical outcomes. uPA patients with a lower urinary sodium-potassium (NaK) ratio were more likely to have clinical success after an adrenalectomy [27]. While urinary NaK ratio is associated with dietary sodium and potassium intake and blood pressure, it also reflects the aldosterone activity. Participants who had a lower sodium intake and a higher intake of potassium-containing salt had a lower rate of cardiovascular events and death than participants with a regular salt intake [28]. In our reasoning, if GRA is a functional genetic disorder, the surgical success rate should be relatively low because the functional chimeric gene remains in the contralateral adrenal gland. However, in this study, we found that 33.3% of GRA-positive individuals achieved complete surgical success. These results imply that GRA status (supposedly a bilateral adrenal condition) could co-exist with a lateralized PA, and that other factors play some significant role(s) in influencing the surgical outcomes of these patients. For example, if a GRA patient carries a non-functional chimeric gene that co-exists with aldosterone-producing-adenoma (APA) or uPA, then the cause of primary aldosteronism would most likely be APA or uPA, but not the GRA chimeric gene. In such circumstances, unilateral adrenalectomy surgery could play a vital role in helping the patient to reach clinical success. In light of this, selected uPA patients harboring GRA should not be a contraindication for an adrenalectomy.

Diagnosing GRA in the heritability of patients suspected of having secondary hypertension is essential because of the increased co-aggregation of major cardiovascular events in the first-degree relatives of PA patients. Our previous study demonstrated that the familial clustering of PA existing in a population-based study supported a genetic susceptibility leading to PA [12]. GRA is inherited in an autosomal dominant inheritance pattern with high penetrance. The early identification of concealed GRA patients would enable the provision of remediable medical therapy to the patients. Given the currently widely available and potent antihypertensive medications, we rarely consider using glucocorticoids with our patients. A few patients could not achieve the target blood pressure control despite using mineralocorticoid receptors and other antihypertensives, in which case glucocorticoids were initiated. We had only six patients in our cohort who needed to use low-dose glucocorticoids. In contrast, tackling the favorable surgical outcomes of GRA patients with a confirmed unilateralized PA would reduce the need for lengthy and exhausting medical treatment.

### 4.2. Factors Related to Hypertension-Remission of GRA Patients after Adrenalectomy

The independent predictive value of serum potassium and PRA illustrated the complex interplay between the potassium and renin–angiotensin–aldosterone axis. Low serum potassium tends to moderate aldosterone hypersecretion in an average individual but potentiates renin secretion. Conversely, high autonomous aldosterone hypersecretion tends to reduce serum potassium levels [29]. PA patients with a high PRA suggested the presence of higher plasma aldosterone levels. Hormonal factors associated with the blood pressure outcome of an adrenalectomy in PA patients have not been consistently proven. A previous report has found that a higher ARR and a urinary aldosterone excretion at baseline were associated with the resolution of hypertension in a univariable, but not a multivariable, analysis [30]. Letavernier et al. reported that urinary aldosterone concentration was associated with curing hypertension after surgery in multivariable analysis [31]. This study found that GRA patients with low serum potassium and high PRA had better surgical outcomes than their counterparts.

### 4.3. Limitation and Strength of the Study

This study represented a selected subgroup of the uPA population from the TAIPAI registry. There were only seven families with GRA in our documented database. We recorded whether the patient had a family history of hypertension or not. However, it is challenging for the family of most GRA patients to sign consent for genetic testing. Thus, such cultural disparity limited us to identifying GRA families in our population. The retrospective nature of our data significantly limited our ability to explain the causal relationship of the identified factors associated with GRA. Long-range PCR confirmed the GRA cases in this study. Thus, the possibility of false-positive GRA status was less likely. We recommend that GRA screening should not be limited to young individuals who have hypertension or stroke.

Moreover, we did not provide the interaction of GRA and present somatic mutations of APA. Thus, we cannot conclude that GRA was the only mutation in the presenting PA population. Lastly, we could not check the cross-over point of each GRA mutation that may be attributed to uneven blood pressure.

## 5. Conclusions

A GRA status could co-exist with a patient with unilateral PA. Older PA patients with better kidney function and plasma aldosterone levels less than 40 ng/dL were more likely to harbor GRA. Individuals with lower serum potassium were associated with better surgical outcomes, independent of the presence of GRA. Additionally, both serum potassium and PRA were significantly associated with hypertension-remission among GRA patients after an adrenalectomy.

## Figures and Tables

**Table 1:** Basic characteristics of GRA-positive and GRA-negative PA patients.

Variables	GRA-Positive (*N* = 79)	GRA-Negative (*N* = 114)	*p* Value
Women/men (%)	39/40 (49.4%/50.6%)	65/49 (57.0%/43.0%)	0.298
Age (years)	51.2 ± 13.8	48.1 ± 13.0	0.081
Body weight (kg)	72.5 ± 17.0	71.0 ± 13.7	0.442
BMI (kg/m^2^)	26.5 ± 4.4	26.1 ± 4.2	0.448
eGFR (MDRD) (ml/min/1.73 m^2^)	92.6 ± 36.7	88.7 ± 29.8	0.338
Na (mmol/L)	140.5 ± 2.9	140.0 ± 3.6	0.450
K (mmol/L)	3.82 ± 0.64	3.79 ± 0.62	0.399
Cholesterol (mg/dL)	180.4 ± 38.8	183.9 ± 34.5	0.562
Triglyceride (mg/dL)	136.7 ± 76.9	133.9 ± 81.1	0.820
LDL-C (mg/dL)	111.6 ± 29.7	108.7 ± 27.4	0.542
TTKG	5.43 ± 2.1	5.49 ± 2.7	0.880
pH	7.41 ± 0.04	7.40 ± 0.03	0.226
HCO_3_	26.3 ± 3.1	25.8 ± 3.6	0.601
Cortisol (μg/dL)	11.8 ± 6.0	11.4 ± 4.7	0.740
SBP (mmHg)	156 ± 20	152 ± 22	0.215
DBP (mmHg)	94 ± 15	92 ± 15	0.591
Intact PTH (pg/mL)	67.01 ± 34.23	61.48 ± 31.54	0.122
Cystatin C (mg/L)	0.75 ± 0.13	0.85 ± 0.31	0.112
CRP (mg/dL)	0.27 ± 0.47	0.42 ± 0.92	0.329
PAC (ng/dL)	41.8 ± 30.9	55.1 ± 37.6	0.008 **
PRA (ng/mL per hour)	0.86 ± 1.46	0.80 ± 1.32	0.469
ARR (ng/dL per ng/mL-h)	364.6 ± 720.4	872.0 ± 1968.0	0.013 *
Urine Aldo	19.1 ± 14.7	15.8 ± 18.4	0.271
ACR (μg/mg)	0.13 ± 0.43	0.19 ± 0.92	0.622
Diabetes	15 (19.0%)	18 (15.8%)	0.552
MACE	12 (15.2%)	8 (7.0%)	0.119
Number of Anti-HTN drugs	3 (0–8)	3 (0–8)	0.900
Family member of HTN	2 (1–3)	1 (1–3)	0.831

Abbreviations: ACR, urine albumin/creatinine ratio; ARR, aldosterone renin ratio; CRP, C-reactive protein; DBP, diastolic blood pressure; eGFR (MDRD), estimated glomerular filtration rate (Modification of Diet in Renal Disease formula); family member of HTN, family history of hypertension; intact PTH, intact parathyroid hormone; K, potassium; MACE, major adverse cardiovascular event; number of anti-HTN drugs, number of antihypertensives drugs in use; Na, sodium; PAC, plasma aldosterone; PRA, plasma renin activity; urine aldo, urine aldosterone; SBP, systolic blood pressure; TTKG, transtubular potassium gradient. * *p* < 0.05, ** *p* < 0.01.

**Table 2:** Univariable and multivariable logistic regression analysis of associated clinical and biochemical factors for GRA patients.

Variables	Univariable		Multivariable	
	Odds Ratio (95% CI)	*p* Value	Odds Ratio (95% CI)	*p* Value
Age (years)	1.02 (1.00–1.04)	0.075	1.02 (1.00–1.04)	0.005 **
eGFR (MDRD) (mL/min/1.73 m^2^)	1.01 (0.98–1.02)	0.347	1.01 (1.00–1.02)	0.001 **
Number of Anti-HTN drugs	1.02 (0.87–1.20)	0.822		
SBP (mmHg)	0.99 (0.98–1.01)	0.221	0.99 (0.97–1.00)	0.079
Intact PTH (pg/mL)	0.99 (0.97–1.01)	0.138		
Cystatin-C (mg/L)	0.17 (0.02–1.71)	0.133		
LnAldo	0.14 (0.05–0.45)	0.001 **	0.16 (0.05–0.51)	0.006 **
PRA	1.03 (0.84–1.27)	0.572		
ARR	1.00 (0.98–1.01)	0.049 *		
ACR	0.89 (0.54–1.47)	0.654		
MACE	2.05 (0.85–4.90)	0.109		

Abbreviations: ACR, urine albumin/creatinine ratio; ARR, aldosterone renin ratio; eGFR (MDRD), estimated glomerular filtration rate (Modification of Diet in Renal Disease formula); intact PTH, intact parathyroid hormone; LnAldo, logarithmic scale of plasma aldosterone; MACE, major adverse cardiovascular event; number of anti-HTN drugs, the number of antihypertensives drugs in use; PRA, plasma renin activity. * *p* < 0.05, ** *p* < 0.01.

**Table 3:** The association of pre-operative variables predicting hypertension-remission (*n* = 97) in unilateral PA patients by logistic regression.

Variable	Estimate	SE.	*p* Value	OR	Lower CI	Upper CI
Age (years)	−0.017	0.025	0.485	0.983	0.936	1.032
Cr	0.521	0.577	0.366	1.684	0.544	5.213
SBP	0.022	0.021	0.291	1.022	0.982	1.064
DBP	−0.024	0.027	0.381	0.976	0.925	1.030
GRA	−0.264	0.562	0.638	0.768	0.255	2.308
Gender	−0.579	0.552	0.294	0.560	0.190	1.654
K	−1.257	0.472	0.008	0.285	0.113	0.717
PRA	0.541	0.298	0.069	1.718	0.959	3.077
PAC	−0.008	0.009	0.394	0.992	0.975	1.010

Abbreviations: Cr, creatinine; DBP, diastolic blood pressure; K, potassium; PAC, plasma aldosterone; PRA, plasma renin activity; SBP, systolic blood pressure.

**Table 4:** The association of pre-operative variables predicting hypertension-remission in GRA patients (N = 36) after adrenalectomy by logistic regression.

Variable	Estimate	SE.	*p* Value	OR	Lower CI	Upper CI
Age (years)	1.169	1.178	0.321	3.217	0.320	32.341
Cr	0.218	0.884	0.806	1.243	0.220	7.031
SBP	−1.124	1.309	0.390	0.325	0.025	4.224
DBP	0.009	0.042	0.828	1.009	0.930	1.095
Gender	−0.592	1.070	0.580	0.553	0.068	4.503
K	−2.019	1.018	0.048	0.133	0.018	0.978
PRA	2.455	1.227	0.045	11.645	1.052	128.940
PAC	−0.016	0.021	0.457	0.984	0.945	1.026

Abbreviations: Cr, creatinine; DBP, diastolic blood pressure; K, potassium; PAC, plasma aldosterone; PRA, plasma renin activity; SBP, systolic blood pressure.

## Data Availability

The data are all included in the manuscript or can be acquired from the corresponding author.

## Supplementary Materials

The following are available online at https://www.mdpi.com/article/10.3390/biomedicines9121816/s1: Figure S1–Long-range PCR of CYP11B1/CYP11B2 chimeric gene. Long-range PCR was conducted to analyze CYP11B1/CYP11B2 chimeric gene in blood genomic DNA of the patients. The expected size of the chimeric CYP11B1/CYP11B2 chimeric gene is 3.9-kb [16]. Using primer set for CYP11B2 with the same long-range PCR reaction produced a 4.0-kb control, CYP11B2 bands. On the left panel, the chimeric gene, lanes 3, 5, and 7, represent the 3.9-kb chimeric bands in GRA-positive individuals. The right panel, CYP11B2 gene, lanes 1–7, represents the uncropped gel electrophoresis image of the control CYP11B2 product. Primer set of CYP11B2 gene as per our report was: Forward sequence 5′CAGGTCCAGAGCCAGTTCTCCCAT and reverse sequence 5′ ACCCTCCTTCTCCTTGTACACCCA. Primer set of the chimeric CYP11B1/CYP11B2 gene as per our report was: Forward sequence 5′CAGTTCTCCCATGACGTGATCCCT and reverse sequence 5′ACCCTCCTTCTCCTTGTACACCCA.
