# Supplementary material for: Characteristics and Outcomes in Primary Aldosteronism Patients Harboring Glucocorticoid-Remediable Aldosteronism
Source: Biomedicines. 2021 Dec 2;9(12):1816. doi: 10.3390/biomedicines9121816 (PMC8698750; doi:10.3390/biomedicines9121816)
Supplement: Supplementary file 1 [file biomedicines-09-01816-s001.zip › biomedicines-1435829-supplementary.pdf]

biomedicines

MDPI

Article

Characteristics and Outcomes in Primary Aldosteronism Patients Harboring Glucocorticoid-Remediable Aldosteronism

Chung-Yi Cheng [1,2,3], Hung-Wei Liao [4], Kang-Yung Peng [5], Tso-Hsiao Chen [1,2,3], Yen-Hung Lin [5], Jeff S. Chueh [6], Vin-Cent Wu [5,*] and on behalf of TAIPAI Study Group [†]

1. Division of Nephrology, Department of Internal Medicine, School of Medicine, College of Medicine, Taipei Medical University, No. 250 Wu-Hsing Street, Taipei 110, Taiwan; 94426@w.tmu.edu.tw (C.-Y.C.); 88128@w.tmu.edu.tw (T.-H.C.)
2. Division of Nephrology, Department of Internal Medicine, Wan Fang Hospital, Taipei Medical University, No 111 Section 3, XinLong Road, Taipei 116, Taiwan
3. Taipei Medical University-Research Center of Urology and Kidney (RCUK), School of Medicine, College of Medicine, Taipei Medical University, No 250 Wu-Hsing Street, Taipei City 110, Taiwan
4. Chinru Clinic, Taipei 116, Taiwan; lhw898@gmail.com
5. Department of Internal Medicine, National Taiwan University Hospital, Room 1555, Clinical Research Building. 7 Chung-Suan South Road, Taipei 100, Taiwan; kangyung@ntu.edu.tw (K.-Y.P.); yenhunglin@ntuh.gov.tw (Y.-H.L.)
6. Department of Urology, College of Medicine, National Taiwan University, National Taiwan University Hospital, Taipei 110, Taiwan; scchueh@ntu.edu.tw

* Correspondence: q91421028@ntu.edu.tw; Tel.: +886-2-23123456 (ext. 63098)

† TAIPAI, Taiwan Primary Aldosteronism Investigation (TAIPAI) Study Group, Taipei, Taiwan.

Long-range PCR- CYP11B1&B2 (patients' blood genomic DNA)

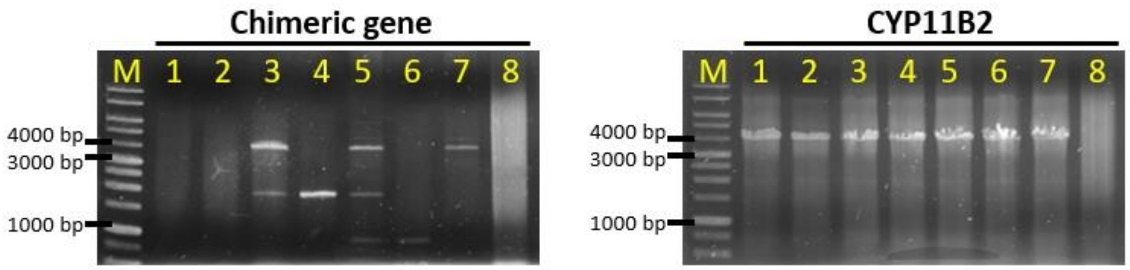

Figure S1. Long-range PCR of CYP11B1/CYP11B2 chimeric gene. Long-range PCR was conducted to analyze CYP11B1/CYP11B2 chimeric gene in the blood genomic DNA of the patients. The expected size of the chimeric CYP11B1/CYP11B2 chimeric gene is 3.9-kb[1]. Using primer set for CYP11B2 with the same long-range PCR reaction produced a 4.0-kb control, CYP11B2 bands. The chimeric gene, lanes 3, 5, and 7 represent the 3.9-kb chimeric bands in GRA-positive individuals on the left panel. The right panel, CYP11B2 gene, lane 1-7, represents the uncropped gel electrophoresis image of the control CYP11B2 product. .

Primer sets of CYP11B2 gene and the chimeric CYP11B1/CYP11B2 gene as our report was, CYP11B2: Forward sequence: 5'CAGGTCCAGAGCCAGTTCTCCCAT/Reverse sequence: 5'ACCCTCCTTCTCCTTGTACACCCA; CYP11B1/CYP11B2: Forward sequence: 5'CAGTTCTCCCATGACGTGATCCCT /Reverse sequence: 5'ACCCTCCTTCTCCTTGTACACCCA.

Reference:

1. Lin, Y.F.; Peng, K.Y.; Chang, C.H.; Hu, Y.H.; Wu, V.C.; Chueh, J.S.; Wu, K.D. Adrenalectomy Completely Cured Hypertension in Patients With Familial Hyperaldosteronism Type I Who Had Somatic KCNJ5 Mutation. *J Clin Endocrinol Metab* **2019**, *104*, 5462-5466, doi:10.1210/jc.2019-00689.
